# Hyper- and hypo- nutrition studies of the hepatic transcriptome and epigenome suggest that PPARα regulates anaerobic glycolysis

**DOI:** 10.1038/s41598-017-00267-9

**Published:** 2017-03-14

**Authors:** Anthony R. Soltis, Shmulik Motola, Santiago Vernia, Christopher W. Ng, Norman J. Kennedy, Simona Dalin, Bryan J. Matthews, Roger J. Davis, Ernest Fraenkel

**Affiliations:** 1 Department of Biological Engineering, Massachusetts Institute of Technology, Cambridge, MA 02139 USA; 2 Howard Hughes Medical Institute and Program in Molecular Medicine, University of Massachusetts Medical School, Worcester, MA 01605 USA

## Abstract

Diet plays a crucial role in shaping human health and disease. Diets promoting obesity and insulin resistance can lead to severe metabolic diseases, while calorie-restricted (CR) diets can improve health and extend lifespan. In this work, we fed mice either a chow diet (CD), a 16 week high-fat diet (HFD), or a CR diet to compare and contrast the effects of these diets on mouse liver biology. We collected transcriptomic and epigenomic datasets from these mice using RNA-Seq and DNase-Seq. We found that both CR and HFD induce extensive transcriptional changes, in some cases altering the same genes in the same direction. We used our epigenomic data to infer transcriptional regulatory proteins bound near these genes that likely influence their expression levels. In particular, we found evidence for critical roles played by PPARα and RXRα. We used ChIP-Seq to profile the binding locations for these factors in HFD and CR livers. We found extensive binding of PPARα near genes involved in glycolysis/gluconeogenesis and uncovered a role for this factor in regulating anaerobic glycolysis. Overall, we generated extensive transcriptional and epigenomic datasets from livers of mice fed these diets and uncovered new functions and gene targets for PPARα.

## Introduction

Diet plays a significant role in shaping human health and disease. Over nutrition leading to obesity can induce insulin resistance, a major human health concern that promotes the development of type 2 diabetes and some cancers^1–3^. In contrast, caloric restriction can extend lifespan, improve insulin sensitivity, and delay the onset of age-related diseases, such as diabetes, cardiovascular disease, and neoplasia^4, 5^. While the broad contrasts between high-fat diet feeding and calorie restriction are well established, the underlying molecular processes that drive these physiological and metabolic differences are incompletely understood.

The liver is a critical regulator of metabolism and is sensitive to dietary changes. The liver maintains normal glucose homeostasis by suppressing hepatic gluconeogenesis in response to insulin following feeding, while promoting glucose production during fasting^6, 7^. High-fat diet induced obesity and insulin resistance disrupts these hepatic mechanisms and promotes hyperglycemia^8^. Caloric restriction, however, lowers liver fat accumulation and improves hepatic glucose regulation in obese humans^9, 10^ and reduces the expression of stress and inflammatory genes in mouse livers, which may contribute to the anti-aging effects associated with this diet^11^. The liver, therefore, is a critical driver of the body’s response to dietary challenges. Thus, analysis of hepatic responses to dietary extremes may enhance our understanding of how diet shapes overall human health.

In this study, we profiled transcriptional and epigenomic landscapes in the livers of mice fed either a standard laboratory chow diet (CD), a long-term (16 week) high-fat diet (HFD) to induce obesity and insulin resistance, or a nutrition-restricted diet to model caloric restriction (CR). Overall, we present a comprehensive analysis of diet-induced effects on mRNA expression and chromatin accessibility in the mouse liver following HFD and CR. We found that calorie restriction and high fat feeding have common and independent epigenetic and transcriptomic signatures. We also show that PPARα activation underlies both extreme metabolic situations and identify new PPARα targets that regulate glucose metabolism.

## Results

### High-fat diet and calorie restriction induce extensive changes in hepatic gene expression

**Figure 1:**
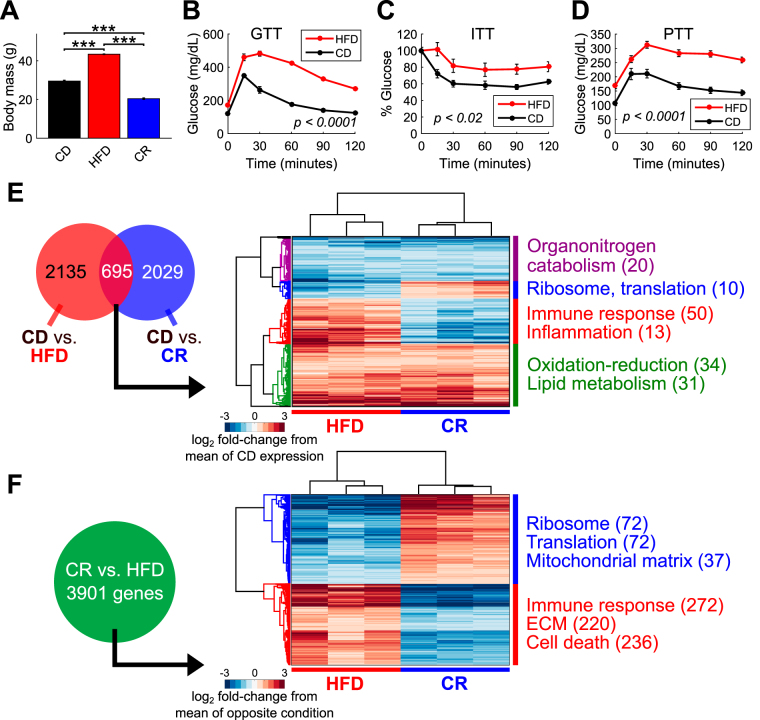
High-fat diet and calorie restriction alter body mass and induce extensive hepatic transcriptional changes. (**A**) HFD and CR feeding increase and decrease overall mouse body mass, respectively, compared to CD (n = 12, 10, and 12 for CD, HFD, and CR, ***p < 5e-5, two-sided t-tests). (**B–D**) HFD induces insulin resistance and alters glycemic regulation as assessed by (**B**) glucose tolerance test (GTT), (**C**) insulin tolerance test (ITT), and (**D**) pyruvate tolerance test (PTT) (*p*-values from t-tests of area under the curve measurements, n = 30, 25, and 23 for CD and n = 37, 27, and 23 for HFD). (**E**) Venn diagrams show numbers of genes differentially expressed between CD and HFD livers (red circle) as well as CD and CR livers (blue circle). The overlap region shows 695 genes that are differentially expressed in both CR and HFD compared to CD. The clustergram shows these 695 overlapping genes that are up-regulated by both HFD and CR (255 genes), down-regulated by both CR and HFD (183 genes), up-regulated in HFD and down-regulated by CR (186 genes), and up-regulated in CR but down-regulated in HFD (71 genes), along with gene ontology and pathway enrichment terms. The numbers indicate how many genes in each group that are annotated to each term. Values are log_2_ fold-changes for individual replicate expression levels (in FPKM) versus the mean CD expression level. (**F**) 3,901 genes are differentially expressed between CR and HFD livers (green circle). The clustergram shows individual replicate gene expression levels as log_2_ fold-change compared to the mean expression level for the opposite condition (CR or HFD). The numbers indicate how many genes in each group that are annotated to each term.

We examined mice following a long-term (16 week) high-fat diet (HFD) or a calorie restricted (CR) feeding protocol. As anticipated, mice fed a HFD gained body mass while CR mice lost mass compared to chow diet (CD)-fed controls (*p* < 5e-5, two-sided t-tests) (Fig. 1A). We assessed glucose homeostasis in HFD mice compared to controls using tolerance tests for glucose (GTT, Fig. 1B), insulin (ITT, Fig. 1C), and pyruvate (PTT, Fig. 1D) and confirmed that mice fed a HFD are strongly insulin resistant and glucose intolerant.

We comprehensively quantified the hepatic transcriptomic landscapes of these mice using RNA-Seq (Fig. S1B and Table S1). Both HFD and CR induced widespread changes in hepatic gene expression compared to CD, with 2,830 and 2,724 genes differentially expressed by the two conditions, respectively (FDR < 0.05, absolute log_2_ fold-change > 0.5) (Fig. 1E). HFD induced the expression of genes involved in immune responses (FDR < 6.4e-22, e.g. *Ccr1*, *Ccr2*, *Cd36*, *Tlr1*), lipid metabolism (FDR < 8e-6, e.g. *Abcd1*, *Apoa4*, *Cyp17a1*, *Srebf1*, *Thrsp*), stress responses (FDR < 1.3e-5, e.g. *Anxa1*, *Axl*, *Car3*, *Hif1a*, *Jak2*), and cell death (FDR < 6e-4, e.g. *Bak1*, *Casp7*, *Jun*), among others. CR up-regulated genes are involved in cholesterol metabolism (FDR < 2.5e-11, e.g. *Cebpa*, *Dhcr7*, *Hmgcr*, *Ldlr*) and mitochondria (FDR < 7e-7, e.g. *Atp5e*, *Cox5a*, *Mrps24*), among other processes.

We found a significant set of 695 genes (*p* < 3.6e-14, hypergeometric test of 695 overlapping genes) that are differentially regulated by both HFD and CR compared to CD, including 255 genes up-regulated by both HFD and CR, 183 down-regulated by both, 186 up-regulated in HFD and down-regulated by CR, and 71 up-regulated in CR but down-regulated in HFD (Fig. 1E and Table S1). Of note, the majority of these genes (438 or ~63%) change in the same direction compared to CD (*p* < 2e-14, Fisher’s exact test). The first set of genes (up-regulated in both conditions) is enriched in processes related to oxidation-reduction (FDR < 0.004) and lipid metabolism (FDR < 0.021). This latter category includes genes involved in cellular fatty acid synthesis (e.g. *Fads1*, *Fads2, Fasn*, *Scd1*), lipid and cholesterol production (*Dhcr24*, *Nsdhl*, *Srebf1*), triglyceride synthesis (*Thrsp*), and peroxisomal import of free fatty acids (*Abcd1*, *Abcd2*). We note that expression changes in oxidation-reduction and lipid metabolism in CR mice are not a consequence of any increases in consumed dietary fat content, as the CR diet contains a similar percentage fat content to the CD and because CR mice consumed overall less food, and therefore less fat, than both the CD and HFD mice. The second set of overlapping genes (down-regulated in both conditions) is enriched in organonitrogen catabolism (FDR < 0.02, e.g. *Aass*, *Agxt*, *Cbs*, *Kynu*, *Pnp*). Genes up-regulated by HFD but down-regulated by CR are involved in immune response (FDR < 6.9e-8, e.g. *Apoa4*, *C1qa,b,c*, *Gas6*) and inflammation (FDR < 6.5e-3, e.g. *Aif1*, *Axl*, *Csf1*, *Tgfb1*), while genes up-regulated by CR but down-regulated by HFD compared to CD are involved in translation and ribosomal composition (FDR < 0.019, e.g. *Rpl37*, *Rps15a*, *Rps28*, *Rps3*). This analysis highlights a common set of genes altered by both conditions that, in a majority of cases, are altered in the same way, a surprising result given the differences in the overall metabolic states of CR and HFD mice.

We next compared the CR and HFD liver RNA-Seq samples to directly contrast the two dietary extremes. We found in total 3,901 differentially expressed genes, with 1,857 genes up-regulated in HFD and 2,044 up-regulated by CR (Fig. 1F and Table S1; Fig. S1A for qPCR validation of select genes). Similar to our other comparisons of the gene sets altered by these diets, genes up-regulated by CR are enriched in processes related to ribosomes, mitochondria, translation, and tRNA processing, while HFD-induced genes are enriched in immune responses, extracellular matrix components, and cell death. Thus, although we found evidence for genes regulated similarly following CR and HFD (Fig. 1E), in general these two dietary extremes induce distinct gene expression programs.

### DNase-Seq and motif analyses identify PPARα and RXRα as common regulators of HFD and CR-induced gene expression in liver

**Figure 2:**
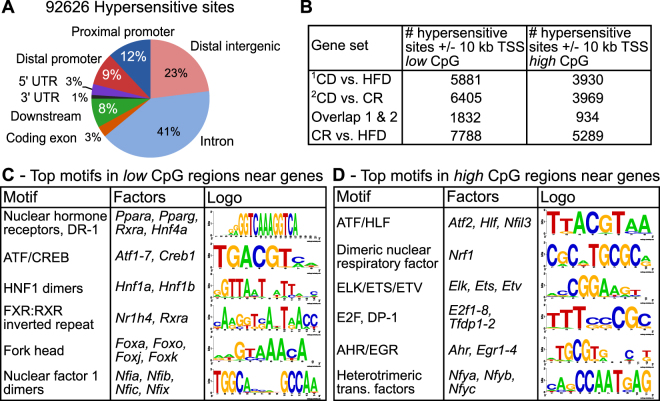
DNase-Seq reveals regulatory regions across liver genomes and motif analyses identify potential transcriptional regulators. (**A**) We found 92,626 hypersensitive regions among CD, HFD, and CR livers. The majority of identified sites reside within annotated gene introns (41%) as well as other near-gene locations. Regions were mapped near genes according to: proximal promoters – within 200 bp of gene TSS; distal promoters – within 5 kb upstream of gene; downstream – within 5 kb downstream of gene end; introns, exons, 5′ UTR, and 3′ UTR – if region intersected one of these features; and distal intergenic – outside 5 kb window around gene. (**B**) The numbers of identified hypersensitive regions near differential gene sets (first column) within +/−10 kb of gene transcription start sites (TSS) in low (middle column) and high (right column) CpG content sequence sets are presented. (**C,D**) The most enriched DNA binding motifs (see Table S2) near all gene sets are shown for low (**C**) and high (**D**) CpG content sequences.

Given the widespread hepatic transcriptional changes induced by both HFD and CR feeding, we performed DNase-Seq on the livers of CD, HFD, and CR mice in order to uncover accessible regulatory regions throughout these genomes that likely harbor regulatory proteins associated with the transcription of these differential genes. Globally, we found high correlations (r = 0.76–0.84) between hypersensitive regions identified in the livers of the mice on the three diets (Fig. S2A–C). For subsequent analyses, we merged the regions identified in all three diets into a set of 92,626 total hypersensitive sites to maximize the search space for regulators (Fig. 2A). We mapped each of these regions to known gene coordinates within the mouse genome and found that the majority of these regions reside within introns (41%). Additional near-gene sites include: proximal promoters (12%), distal promoters (9%), sites downstream of gene bodies (8%), coding exons (3%), 5′ UTRs (3%), and 3′ UTRs (1%). The remaining sites map to distal intergenic regions linearly distant from known gene boundaries (23%). Thus, the majority (77%) of identified hypersensitive regions appear in or near annotated gene boundaries throughout the mouse genome.

As specific examples, we found hypersensitive regions across the conditions near the gene *Cyp2b10*, which is a known target of the nuclear hormone receptors CAR and RXR^12, 13^ (Fig. S2D). Additionally, we found a number of hypersensitive sites near and within the introns of the gene *Abca1*, which is a known target of RXR and LXR in the liver^14^ (Fig. S2E). We performed direct motif analysis on the hypersensitive regions near these select genes and indeed found enrichment for the RXR:LXR motif (Fig. S2F). Thus, the use of motif analysis on the hypersensitive regions near genes altered by diet could reveal regulators associated with these changes.

We examined the discovered DNase hypersensitive regions near the transcription start sites (TSSs, +/−10 kb for this analysis) of our differential gene sets for enriched transcriptional regulator motifs (Fig. 2B and Table S2). We divided the hypersensitive sites near these gene sets into low (<0.5) and high (>0.5) CpG content sequences and assessed motif enrichment in both sets of sequences (see Methods). We found distinct motif enrichments in low versus high CpG content hypersensitive sequences, but consistently observed nearly the same motif enrichments (and rankings) across all the gene sets for the two sets of sequences. In low CpG content regions, we observed strong enrichments near all the gene sets for nuclear hormone direct repeat 1 motifs, corresponding to the factors PPARα, PPARγ, RXRα, and HNF4α, as well as ATF/CREB, HNF1 dimer (HNF1α and HNF1β), FXR:RXR inverted repeat, fork head factor (FOXA, FOXO, FOXJ, FOXK, etc. factors), and nuclear factor 1 dimer (NFIA, NFIB, NFIC, and NFIX) motifs (Fig. 2C). In high CpG content regions, we also observed motif enrichment for ATF factors, though with more preference for thymine as opposed to guanine in the second position of the motif (ATF, HLF factors), as well as dimeric nuclear respiratory factor (NRF1), ELK/ETS/ETV, E2F (E2F and DP-1 factors), AHR/EGR, and heterotrimeric transcription factor (NFYA, NFYB, NFYC) motifs (Fig. 2D). We only found modest enrichments for factors when comparing conditions against one another, e.g. low CpG content regions near up-regulated genes in CR versus regions near genes up-regulated in HFD. This observation is likely due to the fact that we saw such strong enrichments for the same factors in low and high CpG content regions regardless of the gene sets tested. Thus, these factors likely play multiple roles in different contexts to regulate the gene expression programs we observed across the various diets. Given the strong enrichment for nuclear hormone receptor motifs in the low CpG content regions we analyzed, we chose to investigate further the genome-wide binding profiles for the factors PPARα and RXRα to examine their roles in regulating CR and HFD hepatic gene expression.

### ChIP-Seq profiling of PPARα and RXRα binding in CR and HFD livers reveals extensive genome-wide regulation and uncovers novel targets

Our motif analysis strongly suggested that PPARα and RXRα, two transcription factors prominently expressed in liver^15, 16^, contribute to the differential expression of genes in the livers of mice fed either a high fat or calorie restricted diet. We also found significant enrichment for a set of 228 known PPARα target genes among all the differential genes (hypergeometric p-values <5e-4)^17^. For example, 27 of the 695 genes differential in both CR and HFD livers compared to CD (Fig. 1E) are among this set of known PPARα targets (*p* < 3.7e-7). We thus used ChIP-Seq with specific antibodies against these factors (Fig. S3A) to profile their genome-wide binding profiles in CR and HFD livers.

As anticipated from our motif analyses, our ChIP-Seq datasets confirmed that both PPARα and RXRα bind extensively near genes in these livers (Fig. S3B and Table S3). Overall, we detected more RXRα binding than PPARα, likely due to the lower obtained sequencing depth from PPARα samples. Over all binding sites for each factor, we detected some form of the PPAR:RXR heterodimer motif (direct repeat 1) in 91% and 90% of PPARα and RXRα regions, respectively; thus, the majority of identified binding sites contain an expected motif for these factors, though ~10% of these sites likely reflect alternative binding mechanisms (e.g. via other DNA-binding co-regulatory proteins). PPARα binding sites mapped to 1,253 and 2,320 annotated genes in CR and HFD, respectively, while RXRα enriched regions mapped 3,381 and 4,767 genes (+/−10 kb window). The genome-wide binding distributions for these factors also closely mirror those observed in our DNase-Seq experiments, with the majority of binding regions located in introns (42–44%) as well as other near-gene regions (Fig. S3B, left and middle columns). 23–32% of all binding sites were classified as distal intergenic. We also searched for regions in which we found proximal binding events for both factors (peak summits within +/−100 bp) and found 2,831 and 8,838 such regions in CR and HFD livers. The genome-wide binding locations for these regions were similar to those observed for the individual factors (Fig. S3B, right column).

**Figure 3:**
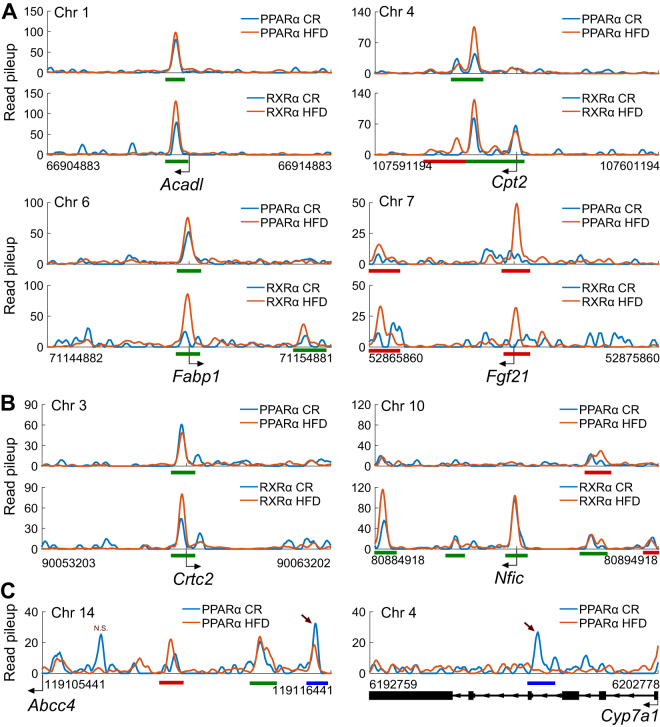
ChIP-Seq of PPARα and RXRα transcription factors in CR and HFD livers reveals extensive binding near known and novel regulated genes. (**A**) The binding profiles (+/−5 kb gene TSS) for known PPARα and RXRα targets *Acadl*, *Cpt2*, *Fabp1*, and *Fgf21* in CR and HFD livers are shown. (**B**) The binding profiles (+/−5 kb gene TSS) for novel PPARα and RXRα targets *Crtc2* and *Nfic* in CR and HFD livers are shown. (**C**) The binding profiles for PPARα near the differentially expressed genes *Abcc4* and *Cyp7a1* (CR vs. HFD) that contain differential binding events between the same two diets in our ChIP-Seq data. Arrows indicate differential binding regions; N.S. stands for not significant. Read pileup refers to extended, normalized, and smoothed read pileup counts extracted from concatenated pools of aligned reads for the biological replicates for each factor (see Methods). Green lines indicate significantly called peaks in both CR and HFD. Red and blue lines indicate significantly called peaks in HFD and CR, respectively.

We used the uncovered binding events to identify known and novel genes that are likely regulated by these factors. PPARα, typically bound as a heterodimer with RXRα, is a well-characterized regulator of lipid metabolism^18^, and we saw strong enrichment for such metabolic processes in up-regulated genes in both CR and HFD livers (Fig. 1E). Consistent with this, we identified binding events near the transcription start sites of genes involved in various lipid metabolic processes which are known to be regulated by PPARα/RXRα^17^, including *Acadl* (involved in mitochondrial β-oxidation), *Cpt2* (involved in mitochondrial oxidation of long-chain fatty acids), *Fabp1* (involved in fatty acid uptake and transport), and *Fgf21* (involved in fatty acid oxidation and ketogenesis) (Fig. 3A). Among these, we found binding evidence for both PPARα and RXRα near *Fgf21* in HFD only (Fig. 3A, bottom right). This result is consistent with our RNA-Seq data in that *Fgf21* is up-regulated in HFD livers compared to CR (log_2_ fold-change of 2.9, FDR < 4.5e-7).

Our analyses identified several novel targets of PPARα and RXRα, including *Crtc2* and *Nfic* (Fig. 3B). *Crtc2* is a known co-regulator of glucose metabolism^19^. We identified binding events for both factors across the two diets at the promoter of this gene. We also highlight binding near *Nfic*, a gene also up-regulated in HFD livers compared to CR, which has up-stream binding events for PPARα in HFD only, as well as clear binding peaks for RXRα alone at its TSS in both CR and HFD. Thus, our profiling of PPARα and RXRα in CR and HFD-fed mouse livers revealed binding events near many genes known to be regulated by these factors, while also uncovering new genes not previously characterized as targets of these factors.

Finally, we tested our PPARα and RXRα ChIP-Seq datasets for evidence of differential binding between CR and HFD livers. We observed a small set of statistically significant differential binding events between the diets for RXRα regions (381 regions, 1.2% of total), even though we identified roughly two times as many called RXRα peaks in HFD compared to CR (Fig. S3B). This result is likely due to thresholding differences during binary peak calling (e.g. due to sequencing depth) which do not always manifest as true statistical differences when comparing read counts in these regions directly. 112 of these 381 differential peaks mapped within +/−20 kb of 103 differential genes between CR and HFD livers. We saw more evidence for differential binding of PPARα between CR and HFD, with 1,201 (9.3% of total) identified peaks showing significant differential enrichment. Only 307 of these, however, mapped to a gene differentially expressed between CR and HFD, covering 284 of the nearly 4,000 potential differential genes. Among these, we observed a differential peak ~10 kb upstream of the *Abcc4* gene promoter that shows lower enrichment in HFD compared to CR (Fig. 3C, left). Indeed, *Abcc4* is expressed significantly lower (~−1 log_2_ fold-change) in HFD compared to CR in our RNA-Seq data. As another example, we found a differential peak with higher enrichment in CR within the gene body of *Cyp7a1*, which is also expressed higher in CR compared to HFD by RNA-Seq (Fig. 3C, right). Though we did not detect many *differential* binding events near these genes, we did detect many binding events in general for these factors near a substantial number of the differential genes. 4,060 PPARα sites map to 1,879 of these genes and 10,271 RXRα peaks map to 2,994. Thus, we found specific instances of differential PPARα and RXRα binding near differential genes between CR and HFD, though such differences only explain small fractions of the total differential gene pools. These results suggest that these factors, given that they indeed bind near many of the differential genes even if the degrees of binding do not measurably change, regulate gene expression differences by mechanisms other than differential binding (e.g. due to differential activity levels or co-factor/co-repressor binding events), though some genes may be more sensitive to differential binding events by other factors.

### mRNA expression, binding data, and fenofibrate-treated primary hepatocytes further suggest a role for PPARα in regulating glucose metabolism

While PPARα has extensively been characterized as a regulator of lipid metabolism, there is evidence that this transcription factor plays a role in regulating glucose metabolism^17, 20–22^. In particular, PPARα knock-out mice show severe hypoglycemia and depleted hepatic glycogen stores during fasting^23^. Moreover, PPARα has been shown to regulate the gluconeogenic genes *G6pc*, *Pck1*, and *Pcx*, the glycerol converting genes *Gpd1* and *Gpd2*, and the pyruvate dehydrogenase inhibitor *Pdk4* ^17, 22, 24^. Indeed, we detected PPARα binding events near the transcription start sites or within the bodies of these genes.

**Figure 4:**
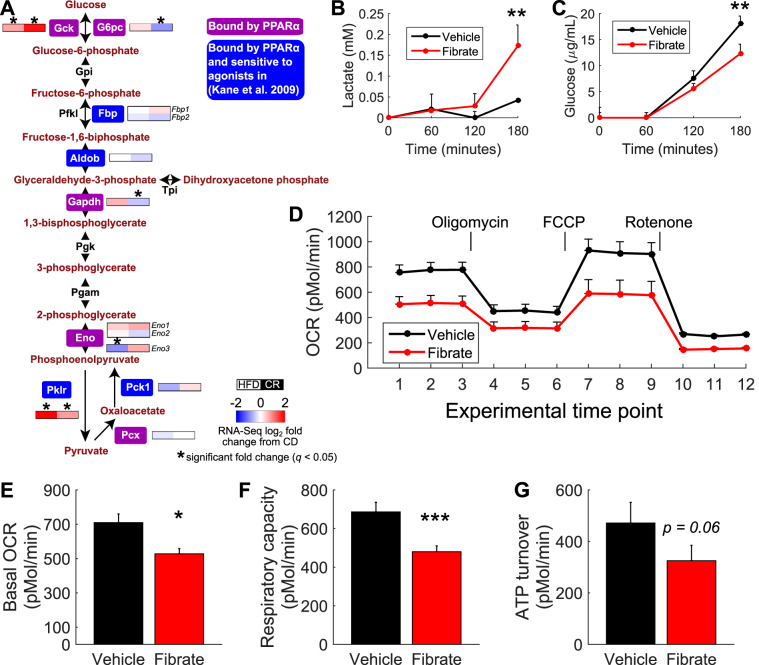
PPARα binds extensively near genes involved in gluconeogenesis/glycolysis in CR and HFD livers and activation by fenofibrate enhances anaerobic glycolysis in primary hepatocytes. (**A**) Canonical gluconeogenesis/glycolysis pathway highlighting genes bound by PPARα (purple outline boxes) and genes both bound by PPARα in our dataset and sensitive to fibrate in Kane *et al*.^20^ (blue outline boxes). The two-element color bars near the bound genes represent the log_2_ fold-changes in mRNA expression (by RNA-Seq) for these genes in HFD and CR livers, respectively, versus CD. *Indicates statistically significant changes (*q* < 0.05). (**B**) Lactate production in mouse primary hepatocytes following vehicle (black line) or fenofibrate (red line) treatment in the presence of glucose. (**C**) Glucose production in the presence of lactate/pyruvate as a gluconeogenic source following vehicle (black line) or fenofibrate (red line) treatment. (**D**) Oxygen consumption rate (OCR) assessed in the presence of glucose following vehicle (black line) or fenofibrate (red line) treatment. OCR also assessed following oligomycin, FCCP, and rotenone drug treatments. (**E–G**) Assessment of basal OCR (**E**), respiratory capacity (**F**), and ATP turnover (**G**) in primary hepatocytes following vehicle or fenofibrate treatment.

We examined genes in the canonical gluconeogenesis/glycolysis pathway for evidence of PPARα binding and found events near nine genes (of fourteen queried) encoding enzymes in this pathway (Fig. 4A). Interestingly, we found that *Aldob*, *Fbp1*, *Fbp2*, *Pck1*, and *Pklr* not only bind PPARα, but are sensitive to PPARα activation^20^ (Fig. 4A, blue highlighted genes). Furthermore, our RNA-Seq data demonstrate PPARα-bound genes are regulated by feeding a HFD or CR, including *Gck* and *Pklr* that are up-regulated by CR and HFD, *G6pc* and *Gapdh* that are down-regulated by CR, and *Eno3* that is down-regulated in HFD (Fig. 4A, colored bars). Thus, PPARα likely influences diet-induced expression changes of these genes.

To further test the role of PPARα in regulating hepatic glucose metabolism, we treated mouse primary hepatocytes with fenofibrate, a PPARα agonist, and measured glycolytic rates. PPARα activation in hepatocytes cultured with glucose as a fuel displayed a significant increase in lactate production, suggestive of an increase in glycolytic flow (Fig. 4B). Consistent with this result, we observed decreased glucose production in the presences of lactate/pyruvate as a gluconeogenic source in fibrate-treated hepatocytes (Fig. 4C). These results suggest that PPARα enhances glycolysis, leading to non-oxidative conversion of glucose to lactate. To test this hypothesis, we assessed the oxygen consumption rate (OCR) in primary hepatocytes using glucose as a fuel (Fig. 4D). OCR was consistently lower in fenofibrate-treated hepatocytes, even in the presence of oxygen consumption inhibitors (oligomycin and rotenone) and enhancers (FCCP). We observed reduced basal OCR (Fig. 4E) and maximal respiratory capacity (Fig. 4F), as well as lower ATP turnover (Fig. 4G), in fenofibrate-treated primary hepatocytes compared to vehicle controls, confirming that PPARα activation decreases oxidative metabolism of glucose. These results, together with our binding data and RNA-Seq results in CR and HFD livers, further stress a role for PPARα in regulating glucose metabolism.

### *In vivo* fenofibrate treatment confirms role of PPARα regulation near genes involved in glucose metabolism in liver

**Figure 5:**
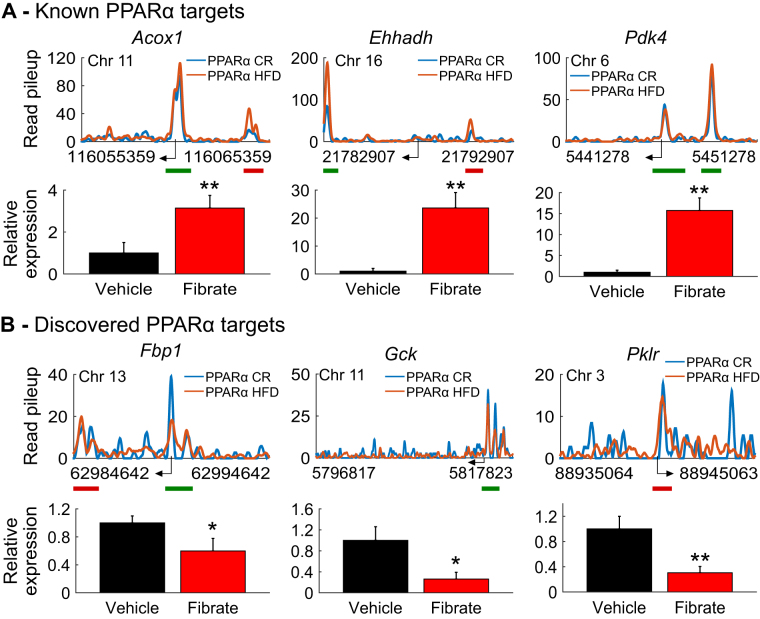
Identified targets are regulated *in vivo* by PPARα. (**A**) Read pileup profiles for CR and HFD PPARα ChIP-Seq near known regulated genes with corresponding *in vivo* qPCR results following fenofibrate treatment. (**B**) Read pileup profiles for CR and HFD PPARα ChIP-Seq near novel regulated genes with corresponding *in vivo* qPCR results following fenofibrate treatment. Read pileup refers to extended, normalized, and smoothed read pileup counts extracted from concatenated pools of aligned reads for the biological replicates for each factor (see Methods). Green lines indicate significantly called peaks in both CR and HFD. Red and blue lines indicate significantly called peaks in HFD and CR, respectively. *p < 0.05, **p *<* 0.01.

We next tested the effect of *in vivo* fenofibrate treatment on specific PPARα targets identified by our ChIP-Seq data. We treated mice for two weeks with either vehicle or fenofibrate and measured hepatic gene expression of PPARα targets using quantitative PCR. We found significant up-regulation of well-characterized PPARα target genes following fibrate treatment, including *Acox1*, *Ehhadh*, and *Pdk4* (Fig. 5A). We next tested several novel PPARα targets identified from our ChIP-Seq data analysis. In keeping with our identification of a role for PPARα in regulating glucose metabolism, we found binding sites near the genes *Fbp1* and *Gck* in both CR and HFD and *Pklr* in HFD. Following *in vivo* fenofibrate treatment, the expression levels of these genes were significantly repressed, providing further support that these are regulated targets of PPARα (Fig. 5B). We also tested other novel targets bound in our ChIP-Seq data, including *Aldh1*, *Aldh2*, *Eno1, Pcx*, and *Sirt3*; however, these were not significantly altered following fibrate treatment by qPCR, suggesting that additional mechanisms are necessary to control their expression *in vivo*.

## Discussion

In this study, we examined the hepatic transcriptional and epigenetic landscapes of mice fed chow, high-fat, and calorie-restricted diets. Joint analysis of our epigenetic data with regulatory protein DNA binding motif data revealed a common set of transcription factors that may regulate the genes altered by these diets. In particular, we found strong enrichments for the PPARα and RXRα motifs near all the identified differential gene sets. We further investigated these findings with direct ChIP-Seq experiments for these factors and found that they do indeed bind extensively near these genes throughout the genomes of HFD and CR mice, further suggesting extensive roles for these factors in the hepatic response to dietary challenges. We particularly focused on the role of PPARα in regulating glucose metabolism in the liver and found extensive binding near genes encoding proteins involved in metabolism of carbohydrates. We validated these findings by treating primary mouse hepatocytes with fenofibrate to stimulate PPARα activation, discovering that activation of this factor enhances anaerobic glycolysis. We also performed *in vivo* fenofibrate treatment experiments in mice and, using quantitative PCR, validated several novel gene targets for PPARα involved in glucose handling. Overall, we present a comprehensive analysis of the effects of high-fat feeding and caloric restriction on mouse hepatic transcriptomics and epigenomics, along with new insights into how the divergent physiological and metabolic states induced by these diets are regulated at the level of transcription.

Our transcriptional profiling data revealed extensive changes in gene expression induced by both HFD and CR compared to CD, as well as many changes between HFD and CR directly. Interestingly, we observed a significant set of 695 genes that change in both extreme diets compared to controls, with 438 of these changing in the same direction. Genes that are up-regulated in both HFD and CR are enriched in oxidation-reduction and lipid metabolic processes, while genes down-regulated in both conditions are enriched in organonitrogen catabolism. Our data thus suggests that some processes and pathways, e.g. fatty acid synthesis, are commonly utilized by the liver in response to divergent dietary challenges. To this end, HFD induces unsaturated fatty acid and triglyceride synthesis to accumulate fat^25^ while CR induces adipose and liver enzymes involved in fatty acid metabolism, including *Fasn* and *Srebf1* which were significantly up-regulated by both diets in our data, to reduce oxidative stress and to induce energy production via β-oxidation^26^. Thus, the liver can co-opt specific pathways for purposes suitable to the needs of various external challenges, in this case dietary changes.

The majority of transcriptional changes induced by HFD and CR, however, are divergent. Specifically up-regulated at the mRNA level in HFD livers are genes involved in immune responses, inflammation, extracellular matrix, and cell death, consistent with anticipated complications resulting from obesity-induced insulin resistance^27–29^. CR livers induced genes related to ribosomes, translational processes, and mitochondria. The inflammatory state observed in HFD livers likely maintains complications related to the insulin resistant state, whereas the reduction in genes associated with these processes in CR, whether compared to CD or HFD, may contribute to the beneficial effects of caloric restriction.

Epigenetic data from DNase-Seq experiments allowed us to map the landscapes of accessible regulatory regions throughout the livers of mice fed each of these diets. We found that most of these accessible regulatory regions reside either within or proximal to known gene boundaries (77%), with the majority residing in introns (41%). We used sequence analysis of these accessible regulatory regions with known DNA-binding motif preferences for regulators to infer factors that are likely associated with transcription of the genes altered by diet. We separated sequences into low and high CpG content sets and looked for motif enrichments across several differential gene sets. While we did find factor enrichment differences between the low and high CpG content sets of sequences, as can be anticipated, we did not find many differences in motif enrichments between the various gene sets. These results suggest that common sets of regulatory proteins are utilized for numerous purposes in the liver. We identified strong enrichments for nuclear hormone receptors, ATF/CREB, and HNF1 factors in low CpG content regions, whereas we found nuclear respiratory factor and ELK/ETS/ETV factor enrichments in high CpG content regions (among others). The strong enrichment of nuclear hormone receptor factors led us to examine the binding profiles for some of these factors more specifically in HFD and CR livers.

We profiled PPARα and RXRα binding throughout the livers of HFD and CR mice using ChIP-Seq. Overall, we found extensive binding for these factors across the genomes as suggested by our motif analyses. We confirmed many known binding sites for these factors near the transcription start sites of specific genes, but also found several novel binding events near genes not known to be regulated by PPARα or RXRα (e.g. *Crtc2* and *Nfic*). We also directly compared binding events for these factors between HFD and CR. Overall, we found that only 1.2% (381 regions) of RXRα binding sites were differential between the diets, whereas a greater percentage (9.3%, 1,201 regions) of identified PPARα binding sites showed some evidence for differential binding. However, only a small portion of these differential sites mapped near genes found to be differentially expressed between HFD and CR, though many of these genes do indeed possess at least some binding evidence for these factors within or near their boundaries. We highlighted *Abcc4* and *Cyp7a1* as examples of genes that change in expression between the diets and that also possess a differential binding region for PPARα nearby.

While PPARα is a well-established regulator of lipid metabolism in the liver^18^, we noted extensive binding for this factor near genes involved in glucose metabolism. Prior studies of PPARα mutant mice^22, 30^, induced activation of PPARα in mice^20, 31^, and others^21, 32^ have also suggested a role for PPARα-dependent regulation of carbohydrate metabolism. Here, we found evidence for PPARα binding near many genes specifically involved in the glycolysis/gluconeogenesis pathway (9 of 14 genes in the canonical pathway analyzed), many of which are sensitive to PPARα agonist treatment according to prior data^20^ and five of which are altered in expression in response to HFD and/or CR according to our RNA-Seq data. To further test the role of PPARα in regulating glucose metabolism, we performed *in vitro* experiments in mouse primary hepatocytes and *in vivo* experiments in mice following fenofibrate treatment. We found that activation of PPARα by fenofibrate enhanced lactate production in the presence of glucose, but decreased glucose in the presence of lactate as a fuel. These results suggest a role for PPARα in enhancing anaerobic glycolysis in the liver. To further test these results, we showed that fenofibrate treatment reduces oxygen consumption rates in hepatocytes. We also found that fenofibrate treatment reduces the expression of the genes *Fbp1*, *Gck*, and *Pklr in vivo*, all of which are novel PPARα-regulated genes identified in this study that are involved in glucose metabolism and contain clear binding sites for PPARα near their transcription start sites. Indeed, evidence for a regulatory role of PPARα on *Gck* expression is somewhat contradictory from previous studies^22^. Fibrate has been shown to decrease its expression in mouse (as we see here), though the PPARα agonist WY14643 does not have the same effect. Moreover, rats possess a PPAR response element (PPRE) near this gene that is activated by LXRα/RXRα and PPARγ/RXRα in luciferase assays, though the role of PPARα in such studies has not been elucidated. Here, we show that PPARα indeed binds near the liver promoter of *Gck* and that the expression of this gene is sensitive to *in vivo* fenofibrate treatment. Overall, our results strongly suggest a role for PPARα in regulating glucose metabolism, in particular anaerobic glycolysis.

## Methods

### Animals and treatments

Calorie restricted male C57BL/6 J mice (5 months of age, 40% calorie restriction^33^, 13.7% calories from fat) were obtained from Charles River Laboratories. Additional male C57BL/6 J mice (stock number 000664, Jackson Labs, Bar Harbor, ME) were fed a standard normal (chow) diet (Prolab Isopro RMH 3000, LabDiet, St. Louis, MO, 14.3% calories from fat) or a high fat diet (HFD) (TD.93075; Harlan Laboratories, South Easton, MA, 54.8% calories from fat) for a period of 16 weeks with free access to food and water. All mice used in this study were housed in a facility accredited by the American Association for Laboratory Animal Care (AALAC). Calorie restricted mice were acclimated in the same animal facility as the chow and HFD mice prior to euthanasia. All experiments were carried out in accordance with guidelines for the use of laboratory animals and were approved by the Institutional Animal Care and Use Committees (IACUC) of University of Massachusetts Medical School and Massachusetts Institute of Technology.

Glucose tolerance tests were performed by intraperitoneal injection of mice with glucose (1 g/kg). Insulin tolerance tests were performed by intraperitoneal injection of mice with insulin (0.5 U/kg). Pyruvate tolerance tests were performed by intraperitoneal injection of mice with pyruvate (1 g/kg). Assays were performed using methods described previously^34^.

We also injected 8 week old C57BL/6 male mice intraperitoneally with the fenofibrate (100 mg/kg), the PPARα antagonist GW6471 (10 mg/kg), or with vehicle (DMSO/Solutol HS15/Sterile water) (10:15:75) three times a week over a two week period.

### RNA-Seq

Total RNA was extracted from the livers of mice (three per dietary condition) fasted overnight using the RNeasy Plus Mini kit (Qiagen, Valencia, CA). mRNA was isolated from DNA-free total RNA using an Illumina mRNA Purification Kit (Illumina, San-Diego, CA). The cDNA library was size-fractionated via gel electrophoresis by cutting a narrow slice (~2 mm, +/−25 bp) of the cDNA lane centered at the 300 bp marker. cDNA from the gel slice was extracted using the Qiagen PCR mini elute kit (Qiagen). The sample was then amplified by PCR using the paired-end primers and amplification reagents supplied with the Illumina ChIP-Seq genomic DNA prep kit. The amplified product was purified using a Qiagen PCR mini elute kit (Qiagen). The library was then used to build clusters on the Illumina flow cell according to the manufacturer’s protocol.

Following sequencing, the raw paired-end reads were aligned to known mouse RefSeq gene transcripts obtained from the UCSC table browser^35^ (accessed on May 19, 2016) and the mouse genome (build mm9) with the splice junction-aware short-read alignment tool TopHat (version 2.1.0)^36^. We restricted TopHat to only align to known transcript splice junctions. We used the Bioconductor package conditional quantile normalization (CQN, version 1.6.0)^37^ to remove systematic biases due to GC-content and gene length coverage and used DESeq2 (version 1.0.18)^38^ to perform differential expression analyses. We considered a gene to be differentially expressed if it possessed an absolute log_2_ fold-change between conditions ≥0.5, an FDR-adjusted p-value (q-value) ≤0.05, and was expressed in at least one tested condition (i.e. ≥0.1 FPKM).

### Clustering and enrichment analyses

All hierarchical clustering was performed with the *clustergram* function in Matlab with Euclidean distance and average linkage. For enrichment analyses, we used custom Matlab code implementing the hypergeometric distribution for enrichment p-value calculations and used the Benjamini-Hochberg FDR procedure to correct for multiple hypotheses.

### Microarray analysis

Raw CEL files from a published microarray study were obtained from the Gene Expression Omnibus, accession number GSE12147^20^. This included data from male C57Bl/6 mice treated with several selective PPARα agonists for 24 hr or 5 days at 1 mg/kg/day or water (vehicle) as control. Samples were background adjusted and normalized using the Bioconductor package *gcrma* and tested for differential expression between conditions using *limma* ^39^ in R.

### DNase-Seq

We performed DNase-Seq on livers from mice fed CD, HFD, or CR according to a previously described protocol^40^. Briefly, liver nuclei were isolated from a pool of 3–4 mice using sucrose based buffer and digested with DNaseI (Promega, Madison, WI). The chromatin was incubated overnight with Proteinase K (Life technologies, Grand Island, NY) at 55 °C. DNA was extracted using phenol chloroform and small DNA fragments were isolated using a sucrose gradient ultracentrifugation followed by a gel size selection step. The DNA fragments were subjected to library preparation and sequencing according to the Illumina protocol.

Sites of DNase cleavage are identified as the 5′ ends of the sequenced short reads from the DNase-Seq assay. We used the GPS algorithm^41^ to identify regions of enriched cleavage compared to a control DNase-Seq assay performed on naked genomic DNA (proteins stripped from the chromatin by phenol-chloroform extraction). GPS builds a probabilistic mixture model to predict the most likely positions of binding events at single-base resolution, requiring an empirical spatial distribution of DNase reads around a typical binding event to build its event detection model. To build the empirical distribution, we identified binding regions from PPARα and RXRα ChIP-Seq data in the same condition, centered in on regions containing known motifs for the protein in question, and summed the DNase read distribution at every base pair in a 600 base pair window around these binding sites. We also performed pairwise comparisons between conditions by submitting both DNase datasets to GPS in multiple condition mode.

### Motif analyses

For DNase hypersensitive sites, we took a 100 bp window around the single base GPS-identified sites for calculation of CpG content and motif matching. We calculated normalized CpG content of sequences using^42, 43^:

$${\rm{Normalized}}\,{\rm{CpG}}=\frac{{\rm{Observed}}\,{\rm{CpGs}}}{({\rm{Expected}}\,{\rm{CpGs}}|{\rm{GC}}\,{\rm{content}})}=\frac{{\rm{Observed}}\,{\rm{CpGs}}}{{({\rm{GC}}{\rm{content}}/2)}^{2}}$$

and divided sequences into low (<0.5) and high (>0.5) CpG content sets based on the bimodality of the empirical CpG content distribution obtained.

For motif analyses, we used a set of 1,588 DNA-binding motifs annotated to human and mouse transcriptional regulatory proteins from release 2013.3 of TRANSFAC^® ^^44^, represented as position-specific scoring matrices (PSSMs). All motifs used were of sufficient total information content (>8 total bits). We extracted the underlying genomic sequences from DNase hypersensitive regions and used TAMO^45^ to store the motif PSSMs, read in sequences, and score the sequences for matches to the motifs. We computed a normalized log-likelihood ratio (LLR) score as *LLR*_*norm*_ = (*LLR* − *LLR*_*min*_)*/*(*LLR*_*max*_ − *LLR*_*min*_) for every *k-*base-pair sub-sequence in the region, where *k* is the length of the motif PSSM. A motif match was called if *LLR*_*norm*_ was greater than or equal to the TRANSFAC^®^-computed minimum false positive matrix similarity score threshold (minFP) for that motif. The maximum matching *LLR*_*norm*_ for each motif in each sequence was retained. Regions with no matches to a given motif were given a score of zero. We also computed motif match scores for sets of equally-sized, GC-content matched background sequences obtained by randomly sampling regions from the mm9 genome.

We used a hypergeometric test to determine enrichment of a motif in the sets of foreground sequences (i.e. DNase regions) compared to matching random background sequences. For such tests, we counted, for a given motif, the number of motif matches in both the foreground and background sets of sequences and compared these values to one another. As many of the motif models are redundant, we used affinity propagation to cluster the motifs, using the pairwise Kullback-Leibler divergence as the similarity metric and a self-similarity parameter of −0.4. This procedure created 284 motif clusters. We post-clustered the motif enrichment results, retaining the result from the most significantly enriched motif in each cluster, and corrected the raw p-values with the Benjamini-Hochberg procedure.

### ChIP-Seq

Following overnight fasting, mice were anaesthetized and the liver was processed as previously described^46^. ChIP experiments were performed on two livers per condition (biological replicates) using antibodies against RXRα (sc-153x, Santa Cruz Biotechnology, Santa Cruz, CA) or PPARα (MAB3890, Millipore, Billerica, MA). We fragmented chromatin with a Covaris S2 sonication machine (Covaris, Woburn, MA) to obtain fragments ranging from 500 to 1000 base pairs. 5 µg of antibody or IgG was incubated with beads for 6 hours before incubating with sonicated chromatin overnight. We then washed the beads, eluted the chromatin, reversed crosslinks for 2 hours, and treated samples with RNase and Proteinase K. We purified the DNA and constructed sequencing libraries using the DNA Sample Kit (Part# 0801-0303, Illumina, San Diego, CA) according to the manufacturer’s instructions. The samples were sequenced on an Illumina GAII/HiSeq sequencing platform and the resulting short reads were aligned against the mm9 reference mouse genome using Bowtie (version 0.12.7)^47^. Enriched genomic regions were identified by MACS (version 1.4)^48^ using an IgG control and the resulting peaks were filtered to have an enrichment p-value of <1e-10. Overlapping peaks between RXRα and PPARα ChIP-Seq datasets were restricted to those whose summits mapped within ±100 bp. Transcription factor binding motifs from the TRANSFAC database were used with the THEME software package^49^ to find enriched motifs in the DNA sequences under the filtered ChIP peaks. For ChIP-Seq read pileup visualizations, we concatenated the aligned sequence reads from biological replicates for each factor in each condition, extracted reads mapping within the specified windows (allowing for only two reads mapping to the exact same location to minimize PCR biases), extended each read in the 3′ direction to a full length of 200 bp, summed the number of extended reads overlapping each base pair within the window, normalized the read count levels to account for sequencing depth differences between samples, and smoothed the read profiles using a moving average filter (120 bp rate). Thus, read pileup axes in Figs 3 and 5 refer to these concatenated, extended, normalized, and smoothed read profiles.

### Primary hepatocytes

We isolated mouse primary hepatocytes with a modified 2-step perfusion method^50^ that uses Liver Perfusion Media and Liver Digest Buffer (Invitrogen)^51^. We seeded cells on plates (pre-coated [1 h] rat tail collagen I [BD Biosciences]) in DMEM supplemented with 4.5 g/L glucose, 10% FBS, 0.2% BSA, 2 mM sodium pyruvate, 2 mM glutamine, 1 μM dexamethasone, 100 nM insulin and 1% penicillin/streptomycin. After attachment (2 h), the medium was removed and the hepatocytes were incubated (22 h) in maintenance medium (DMEM supplemented with 4.5 g/L glucose, 0.2% BSA, 2 mM sodium pyruvate, 2 mM glutamine, 0.1 µM dexamethasone, 1 nM insulin and 1% penicillin/streptomycin). In some cases, we incubated hepatocytes (16 h) with fenofibrate (100 μM, Sigma). The drugs were dissolved in DMSO; control studies were performed by addition of vehicle (DMSO) alone.

We evaluated glucose production by incubating 5.5 × 10^5^ primary hepatocytes in collagen-coated 35 mm wells (6 well plates) with M199 media (Invitrogen) supplemented with 0.5% BSA and 1% penicillin/streptomycin for 18 hours. Cells were then incubated in glucose/glutamine/phenol red-free DMEM (Sigma) supplemented with 3.7 g/L sodium bicarbonate, 2 mM lactate and 20 mM sodium pyruvate for the indicated times. Glucose production in the medium was assessed using the glucose (HK) assay kit (Sigma) and values were normalized to total hepatocyte protein.

We evaluated lactate production by incubating 5.5 × 10^5^ primary hepatocytes in collagen-coated 35 mm wells (6 well plates) with M199 media (Invitrogen) supplemented with 0.5% BSA and 1% penicillin/streptomycin for 18 hours. Cells were then incubated in glucose/glutamine/phenol red-free DMEM (Sigma) supplemented with 1.85 g NaCl, 0.2% BSA, 0.1 µM dexamethasone, 1 nM insulin and 138 mM glucose for the indicated times. Lactate production was measured in the medium using the reconstituted Lactate Reagent (Beckman Coulter) and values were normalized to total hepatocyte protein.

### Oxygen consumption rates

We quantified oxygen consumption rates (OCR) in primary hepatocytes using an XF24 Extracellular Flux Analyzer (Seahorse Bioscience, Billerica, MA) and XF assay kits to measure extracellular flux changes of oxygen and protons. Briefly, primary hepatocytes were plated (4 × 10^4^ cells/well) in collagen-coated XF24-microplates (Seahorse Bioscience). After attachment (2 h), the hepatocytes were transferred to running medium (sodium bicarbonate-free DMEM supplemented with 4.5 g/L glucose, 0.2% BSA, 2% penicillin/streptomycin, 1 nM insulin and 0.1 μM dexamethasone) and incubated at 37 °C in a humidified atmosphere without CO_2_ supplementation. Baseline measurements were performed prior to the addition of substrates (1 g/L glucose, 200 µM palmitate-BSA, or 10 mM lactate/1 mM pyruvate) or inhibitors (1 µM oligomycin, 0.1 µM FCCP, or 100 nM rotenone). Mitochondrial oxygen consumption rates were calculated as the difference between the maximal respiratory rate (in the presence of FCCP) and the respiratory rate after addition of rotenone. Data obtained from 11 independent wells were examined for each condition.

### Quantitative RT-PCR

The expression of mRNA was examined by quantitative PCR using a Quantstudio PCR machine (Life Technologies). TaqMan^®^ assays were used to quantify *Acox1* (Mm01246834_m1), *Ehhadh* (Mm00619685_m1), *Fbp1* (Mm00490181_m1), *Gck* (Mm00439129_m1), *Pdk4* (Mm01166879_m1), and *Pklr* (Mm00443090_m1). The relative mRNA expression was normalized by measurement of the amount of *18S* RNA in each sample using TaqMan^©^ assays (catalog number 4308329; Life Technologies).

## Electronic supplementary material

Supplementary Information

Supplementary Dataset 1

Supplementary Dataset 2
