# Supplementary material for: Hyper- and hypo- nutrition studies of the hepatic transcriptome and epigenome suggest that PPARα regulates anaerobic glycolysis
Source: Sci Rep. 2017 Mar 14;7:174. doi: 10.1038/s41598-017-00267-9 (PMC5428070; doi:10.1038/s41598-017-00267-9)
Supplement: Supplementary file 1 — Supplementary Information [file 41598_2017_267_MOESM1_ESM.pdf]

SUPPLEMENTARY INFORMATION FOR: Hyper- and hypo- nutrition studies of the hepatic transcriptome and epigenome suggest that PPARα regulates anaerobic glycolysis

Anthony R. Soltis[1,3], Shmulik Motola[1,3], Santiago Vernia[2,3], Christopher W. Ng[1], Norman J. Kennedy[2], Simona Dalin[1], Bryan J. Matthews[1], Roger J. Davis[2], and Ernest Fraenkel[1,*]

[1]Department of Biological Engineering, Massachusetts Institute of Technology, Cambridge, MA 02139, USA

[2]Howard Hughes Medical Institute and Program in Molecular Medicine, University of Massachusetts Medical School, Worcester, MA 01605, USA

[3]These authors contributed equally to this work.

[*]Correspondence**:** fraenkel-admin@mit.edu Tel: 617-253-0632

SUPPLEMENATRY FIGURES AND TABLES

FIGURE S1: **qPCR validation of CR versus HFD gene expression changes and RNA-Seq sequence read alignment statistics.**
(A) qPCR validation of gene expression level changes between CR and HFD for genes *Alb, Apoa1, Apoa4, Cidea, Egr1, Fmo3, Fos, Il1rn, Rps14,* and *Sirt3*. (**B**) Total obtained reads, alignment percentages, proper paired mapping percentages, and the percentage of uniquely aligned reads for paired-end reads from CD, HFD, and CR RNA-Seq samples.

FIGURE S2: DNase-Seq dataset correlations and example binding profiles.
(**A-C**) Correlation plots between read counts (tags) from CD, HFD, and CR DNase-Seq datasets: HFD versus CD (**A**), CR versus CD (**B**), and HFD versus CR (**C**). Correlation values are for Pearson correlation coefficients. (**D-E**) Example read pileup tracks from CR and HFD DNase-Seq datasets near genes *Cyp2b10* (**D**) and *Abca1* (**E**) which are known to contain LXRα:RXRα binding sites. Bottom tracks show RXRα profiles from CR and HFD ChIP-Seq samples, confirming binding sites in these hypersensitive regions for expected factors. (**F**) Motif logo for LXRα:RXRα DNA-binding preference that is enriched in DNase-Seq regions.

FIGURE S3: Validation of PPARα and RXRα antibodies and binding locations from ChIP-Seq studies.
(**A**) (Left) Nuclear or cytoplasmic fractions of homogenized CR and HFD livers were assayed by Western blot using anti-RXRα or β-actin primary antibodies. (Right) Whole-cell lysates from CR and HFD livers were immunoprecipitated with PPARα antibody; IP and supernatant were immunoblotted with anti PPARα or β-actin antibodies. (**B**) Genome-wide binding locations of PPARα, RXRα, and overlapping PPARα:RXRα ChIP-Seq peaks with total enrichment region numbers. Regions were mapped near genes according to: proximal promoters – within 200 bp of gene TSS; distal promoters – within 5 kb upstream of gene; downstream – within 5 kb downstream of gene end; introns, exons, 5' UTR, and 3' UTR – if region intersected one of these features; and distal intergenic – outside 5 kb window around gene.

TABLE S3. **ChIP-Seq and DNase-Seq dataset statistics.** Information for PPARα, RXRα, and IgG antibodies, total and unique reads, positive and negative peaks called by MACS, gene annotations (+/- 10 and +/- 1 kb), and replicate numbers is included.

Figure S1

A

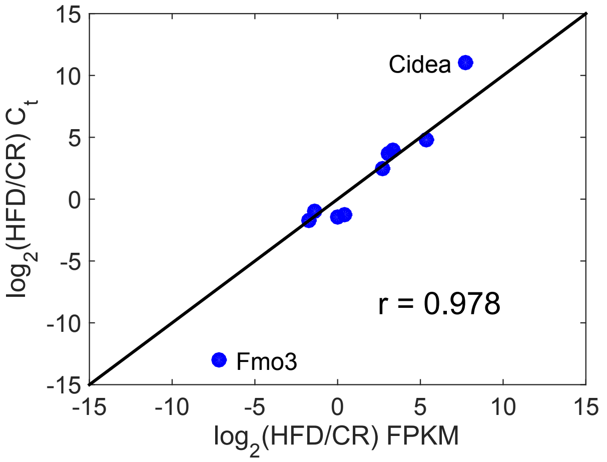

B

**Sample**	**Total paired-end reads**	**% Aligned reads**	**% Proper pair alignments**	**% Aligned uniquely**
CD-1	37643030	97.5	94.1	79.2
CD-2	33414197	96.6	92.4	79.6
CD-3	35945949	96.6	92	79.2
HFD-1	36262557	97.2	93.8	81.3
HFD-2	37447506	96.5	92.8	77.3
HFD-3	25037298	96.6	93.5	84.3
CR-1	39189223	96.6	92.5	85.5
CR-2	19858320	96.9	93.7	85
CR-3	30429450	97.7	94.8	84.7

Figure S2

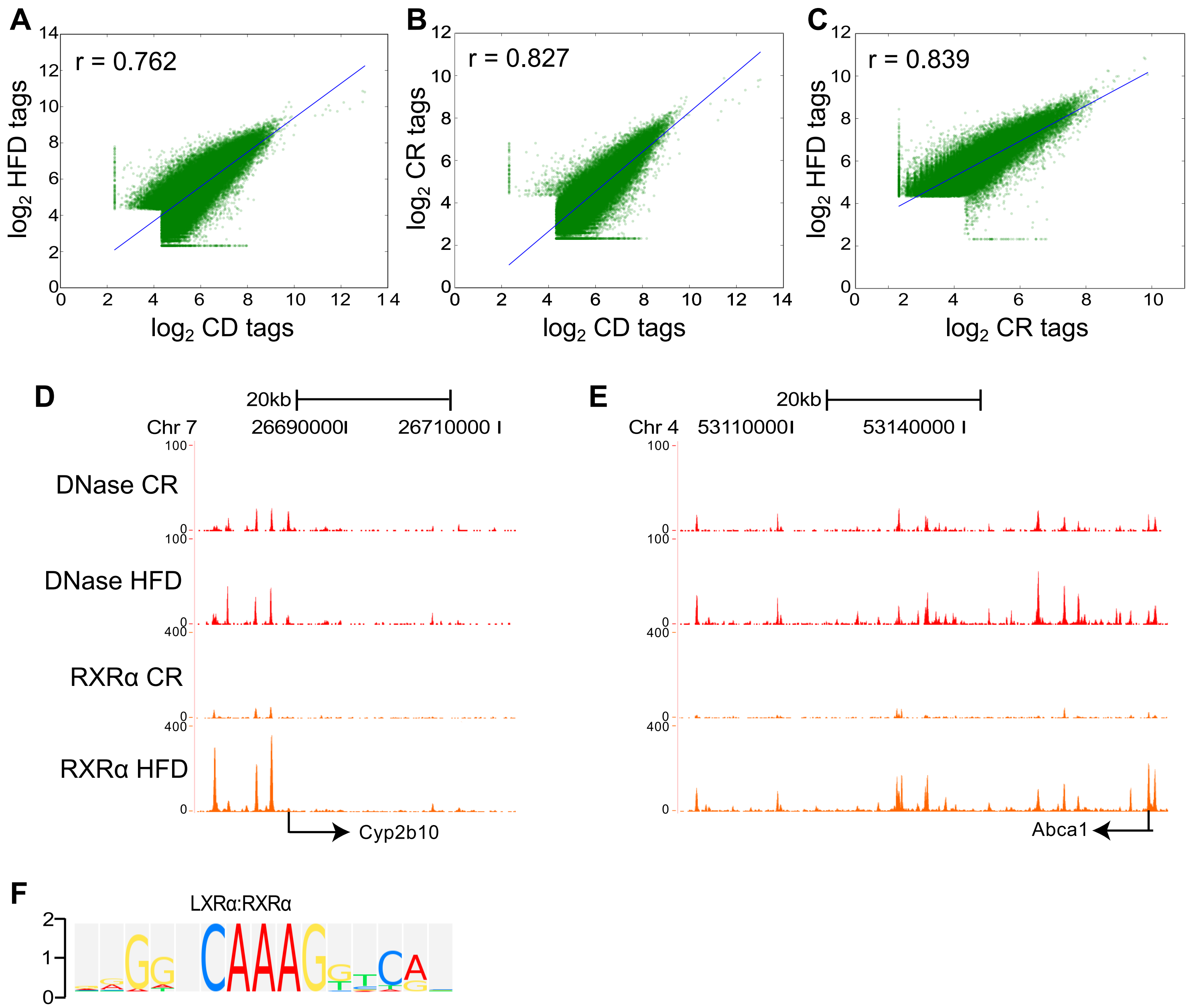

A
B
C
r = 0.762
r = 0.827
r = 0.839
log2 HFD tags
log2 CR tags
log2 HFD tags
log2 CD tags
log2 CD tags
log2 CR tags
D
E
20kb
Chr 7
26690000
26710000
Chr 4
53110000
53140000
DNase CR
DNase HFD
RXRα CR
RXRα HFD
Cyp2b10
Abca1
F
LXRα:RXRα

Figure S3

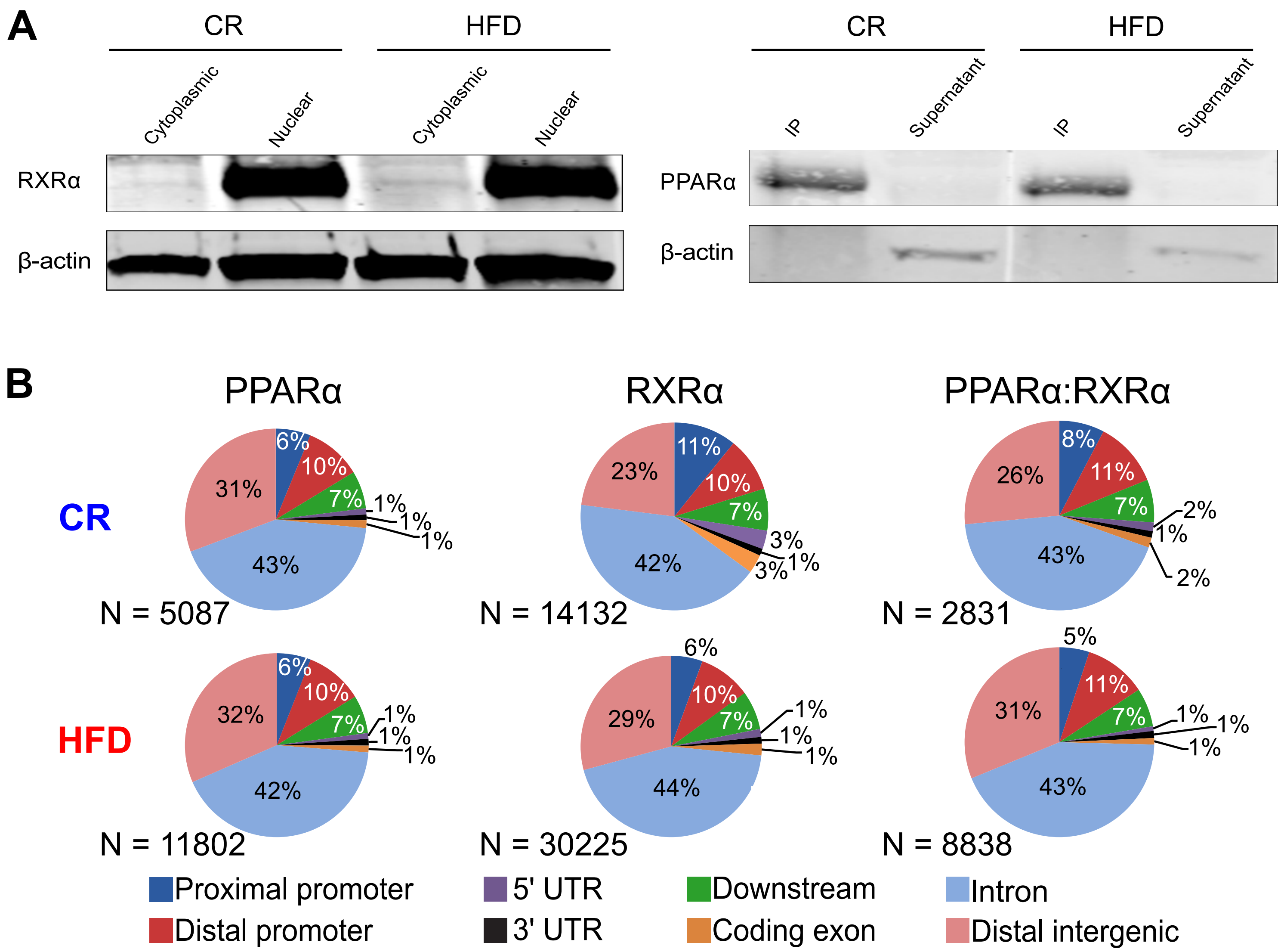

A
CR
HFD
Cytoplasmic
Nuclear
Cytoplasmic
Nuclear
RXRα
β-actin
CR
HFD
IP
Supernatant
IP
Supernatant
PPARα
β-actin
B
PPARα
RXRα
PPARα:RXRα
CR
HFD
N = 5087
N = 14132
N = 2831
N = 11802
N = 30225
N = 8838
Proximal promoter
Distal promoter
5' UTR
3' UTR
Downstream
Coding exon
Intron
Distal intergenic

Table S3

Sample	Cat #	Condition	Number of total reads	Number of unique reads	Positive:negative peaks	Number of annotated genes (+/- 10 kb)	Number of annotated genes(+/- 1 kb)	# of replicates
RXRα	sc-153x	CR	55,118,418	27,591,607	14,132:1,009	3,381	1,887	2 concatenated
RXRα	sc-153x	HFD	51,970,684	37,419,814	30,255:1,922	4,767	2,090	2 concatenated
PPARα	MAB3890	CR	37,853,458	24,214,914	5,087:404	1,253	388	2 concatenated
PPARα	MAB3890	HFD	22,755,500	16,409,143	11,802:450	2,320	808	2 concatenated
IgG	sc-2027	CR	21,225,535	11,052,772				1
IgG	sc-2027	HFD	22,203,246	13,504,158				1
DNase		CD	12,851,994	11,054,890				1
DNase		CR	13,629,774	11,343,038				1
DNase		HFD	13,584,470	11,671,690				1
DNase naked		CD	13,139,144	11,163,521				1
DNase naked		CR	20,579,163	8,030,122				1
DNase naked		HFD	15,368,723	11,324,558				1
